# Glycemic Instability and Skin Health: Metabolic Mechanisms Linking Dietary Patterns with Cutaneous Dysfunction

**DOI:** 10.3390/jcm15155830

**Published:** 2026-07-25

**Authors:** Paulina Cebulla, Emilia Czempik, Hanna Wilk, Iwona Turkowska, Przemysław Domaszewski, Karolina Chilicka-Hebel

**Affiliations:** Faculty of Health Sciences, University of Opole, 45-060 Opole, Poland; paulina.holek@poczta.pl (P.C.); emiliaczempik@gmail.com (E.C.); wilkhanna4@gmail.com (H.W.); iwona.turkowska@uni.opole.pl (I.T.); przemyslaw.domaszewski@uni.opole.pl (P.D.)

**Keywords:** glucose metabolism, skin aging, skin barrier dysfunction, insulin resistance, advanced glycation end products, acne vulgaris

## Abstract

Dietary patterns associated with high glycemic load and recurrent postprandial glucose excursions may influence skin physiology through mechanisms involving oxidative stress, insulin–IGF-1 signaling, chronic low-grade inflammation, and advanced glycation end product (AGE) formation. Disturbances in glucose homeostasis have been associated with alterations in epidermal barrier integrity, sebaceous gland activity, extracellular matrix remodeling, and tissue repair processes. These mechanisms have been investigated primarily in relation to several dermatological conditions, particularly acne vulgaris, impaired wound healing, xerosis, and skin aging. This narrative review summarizes current evidence regarding the relationship between glycemic dysregulation and skin health, with emphasis on metabolic and molecular pathways potentially involved in cutaneous dysfunction. To reflect the current strength of evidence, mechanistic studies, observational studies, and clinical investigations are distinguished throughout this review whenever possible. Particular focus is placed on postprandial glycemic variability, oxidative stress, hormonal signaling, and glycation-related tissue damage. Current evidence supports biologically plausible associations between metabolic dysregulation and several aspects of skin dysfunction, although direct evidence specifically addressing glycemic variability remains limited. Most available studies are observational or mechanistic in nature, and relatively few clinical investigations have evaluated dermatological outcomes directly. Further research is needed to clarify the clinical relevance of glycemic-targeted dietary and metabolic interventions in dermatology.

## 1. Introduction

Nutrition plays an important role in maintaining skin physiology and may influence multiple processes involved in dermatological health, including inflammatory signaling, oxidative balance, hormonal regulation, and tissue regeneration [1,2,3,4].

In recent years, growing attention has been directed toward the relationship between metabolic dysfunction and skin disorders, particularly acne vulgaris, impaired wound healing, xerosis, and premature skin aging [2,3].

Most earlier studies focused mainly on chronic hyperglycemia and diabetes-related skin complications. More recently, researchers have started to investigate whether dynamic disturbances in glucose regulation, such as postprandial glucose spikes, glycemic variability, and compensatory hyperinsulinemia, may also affect skin physiology, even in individuals without diagnosed diabetes mellitus [5,6]. Repeated glucose fluctuations have been associated with increased oxidative stress and activation of pro-inflammatory pathways [7,8]. They have been hypothesized to influence insulin and IGF-1 signaling and to potentially contribute to AGE formation [9,10].

The skin is a metabolically active organ that depends on tightly regulated cellular energy balance to maintain epidermal turnover, barrier integrity, extracellular matrix remodeling, immune defense, and tissue repair. Keratinocytes and dermal fibroblasts may be particularly sensitive to metabolic stress, which may be associated with changes in cellular proliferation, inflammatory signaling, and regenerative capacity [11]. As a result, disturbances in glucose homeostasis may also influence structural and functional aspects of the skin, although much of the available evidence derives from studies of diabetes and chronic hyperglycemia rather than glycemic variability itself [10,11,12,13].

Dietary patterns characterized by high glycemic load and extensive food processing may be particularly relevant in this context. The glycemic impact of diet is determined not only by the glycemic index of individual foods but also by glycemic load, food processing, meal composition, and inter-individual metabolic responsiveness. Classic and updated glycemic index tables provide a framework for estimating carbohydrate quality, whereas CGM-based studies indicate substantial interpersonal variability in postprandial glucose responses [14]. Frequent postprandial glucose elevations have been linked to hyperinsulinemia, oxidative stress, and chronic low-grade inflammation associated with metabolic dysfunction [15,16]. In dermatology, these mechanisms have been investigated most extensively in acne vulgaris, where high-glycemic dietary patterns have been associated with increased IGF-1 signaling, sebaceous gland activity, and greater acne severity [17,18]. At the same time, direct evidence specifically linking glycemic variability with skin dysfunction remains limited. Although glycemic variability is the primary focus of this review, direct dermatological studies evaluating CGM-derived measures remain scarce. Consequently, much of the available evidence discussed herein is derived from mechanistic studies, investigations of chronic hyperglycemia, diabetes mellitus, insulin resistance, or dietary glycemic load that provide biologically plausible insights into the potential effects of glycemic variability [19].

This narrative review evaluates current evidence regarding the relationship between glycemic instability and skin health, with particular focus on metabolic and molecular mechanisms potentially linking dietary patterns, glucose dysregulation, and cutaneous dysfunction. Because direct studies examining glycemic variability and dermatological outcomes remain limited, this review also discusses mechanistic and clinical evidence derived from studies of chronic hyperglycemia, dietary glycemic index/load, insulin resistance, and diabetes, where these findings may provide biologically plausible insights into the potential effects of glycemic variability. Whenever possible, mechanistic, observational, and clinical evidence are discussed separately to reflect the overall strength of the available evidence.

## 2. Materials and Methods

This narrative review was conducted to summarize and critically evaluate the current evidence regarding the relationship between glycemic instability and skin health, with particular emphasis on the metabolic and molecular mechanisms potentially linking disturbances in glucose homeostasis with cutaneous dysfunction.

A literature search was performed in the PubMed and Google Scholar databases between 8 January and 10 July 2026. These databases were selected because they provide comprehensive coverage of biomedical, clinical, nutritional, and interdisciplinary literature relevant to glucose metabolism and dermatology. Publications available from database inception until the final search date were considered, with particular emphasis placed on studies published within the last five years. Earlier landmark publications were included when they provided essential mechanistic background. The search strategy combined Medical Subject Headings (MeSH), where applicable, and free-text terms related to glucose metabolism and skin biology. The search strategy included the following representative search terms: glycemic variability, glycemic instability, postprandial glucose, continuous glucose monitoring, glucose metabolism, glycemic index, glycemic load, insulin resistance, hyperglycemia, oxidative stress, advanced glycation end products, skin aging, skin barrier, xerosis, wound healing, acne vulgaris, cutaneous inflammation, and dermatology. Boolean operators (AND/OR) were used to combine search terms.

Original research articles, observational studies, clinical trials, experimental studies, systematic reviews, and high-quality narrative reviews published in English were considered eligible for inclusion. Studies were included if they evaluated associations between glucose dysregulation (including glycemic variability, postprandial glucose excursions, chronic hyperglycemia, insulin resistance, dietary glycemic index or glycemic load) and skin physiology or dermatological disorders, or if they investigated molecular mechanisms relevant to these relationships. Publications unrelated to skin biology, articles without sufficient methodological information, conference abstracts, editorials, and non-English publications were excluded.

Titles and abstracts were screened for relevance, followed by full-text assessment of potentially eligible articles. Additional studies were identified through manual screening of the reference lists of selected publications. Because direct studies examining glycemic variability and dermatological outcomes remain limited, mechanistic and clinical evidence derived from studies of chronic hyperglycemia, diabetes mellitus, dietary glycemic index, glycemic load, and insulin resistance was also included to provide biological context for the proposed mechanisms and to address gaps in the currently available evidence.

The findings were synthesized narratively and organized according to the principal biological pathways linking glucose dysregulation with skin function, including oxidative stress, insulin–IGF-1 signaling, advanced glycation end product (AGE) formation, epidermal barrier dysfunction, and extracellular matrix remodeling, followed by discussion of their potential relevance to selected dermatological conditions. Particular attention was paid to distinguishing evidence derived from mechanistic studies, observational studies, and clinical investigations in order to reflect the overall strength of the available evidence.

As this review was based exclusively on previously published literature, ethical approval and informed consent were not required.

Generative artificial intelligence (GenAI) tools were used solely for minor language editing and stylistic refinement. Literature selection, critical appraisal of the evidence, interpretation of findings, and scientific conclusions were performed independently by the authors.

## 3. Mechanisms Linking Glycemic Instability with Skin Dysfunction

Although this review focuses on glycemic instability, much of the currently available evidence derives from studies investigating chronic hyperglycemia, diabetes mellitus, or dietary glycemic index and glycemic load. Where direct evidence regarding glycemic variability is unavailable, mechanistic findings from these related metabolic conditions are discussed because they may provide mechanistically plausible pathways linking glucose dysregulation with skin dysfunction.

### 3.1. Glycemic Instability, Oxidative Stress, and Inflammatory Signaling

Glycemic instability refers to recurrent fluctuations in blood glucose concentrations, including postprandial glucose excursions, intermittent hyperglycemia, and rapid shifts in glucose availability associated with impaired metabolic regulation. In contemporary clinical and research settings, glycemic instability is most commonly assessed using continuous glucose monitoring (CGM), which enables near-continuous measurement of interstitial glucose concentrations and provides substantially more detailed information than conventional markers such as glycated hemoglobin (HbA1c) [20,21,22]. Whereas HbA1c reflects average glycemic exposure over approximately three months, CGM captures the frequency, magnitude, duration, and temporal patterns of glucose excursions, allowing direct assessment of dynamic glucose fluctuations [22,23,24].

To facilitate standardized interpretation of glucose profiles, international expert consensus recommends several CGM-derived metrics, including Time in Range (TIR), Time Above Range (TAR), Time Below Range (TBR), coefficient of variation (CV), mean amplitude of glycemic excursions (MAGE), continuous overall net glycemic action (CONGA), and standard deviation (SD). Together, these complementary measures provide a multidimensional assessment of glycemic variability that cannot be obtained using HbA1c or intermittent glucose measurements alone. Among these metrics, the coefficient of variation (CV) is currently regarded as the preferred standardized measure of glycemic variability, whereas TIR has emerged as a key indicator of overall glycemic control in both clinical practice and research [21,22,24,25,26]. These standardized metrics have become the preferred approach for quantifying glycemic variability in both clinical practice and research, replacing reliance on HbA1c alone when dynamic glucose fluctuations are of interest [25].

From the perspective of dermatological research, CGM-derived metrics may provide a valuable methodological framework for investigating whether repeated glucose excursions contribute to skin dysfunction independently of chronic hyperglycemia. Given that glycemic variability represents the central concept of this review, understanding its standardized assessment is essential for interpreting the available evidence and identifying current gaps in dermatological research [21,22,24,25]. Many of the mechanistic pathways discussed throughout this review, including oxidative stress, inflammatory signaling, insulin–IGF-1 dysregulation, AGE formation, epidermal barrier dysfunction, and extracellular matrix remodeling, are hypothesized to be influenced not only by sustained hyperglycemia but also by dynamic fluctuations in glucose concentrations. However, despite the growing availability of CGM technologies, direct studies evaluating CGM-derived measures in relation to skin physiology or dermatological diseases remain scarce. Consequently, the following sections focus primarily on biologically plausible mechanistic pathways supported by experimental and indirect clinical evidence, whereas direct dermatological evidence remains limited [20,21,22,24].

**Figure 1:**
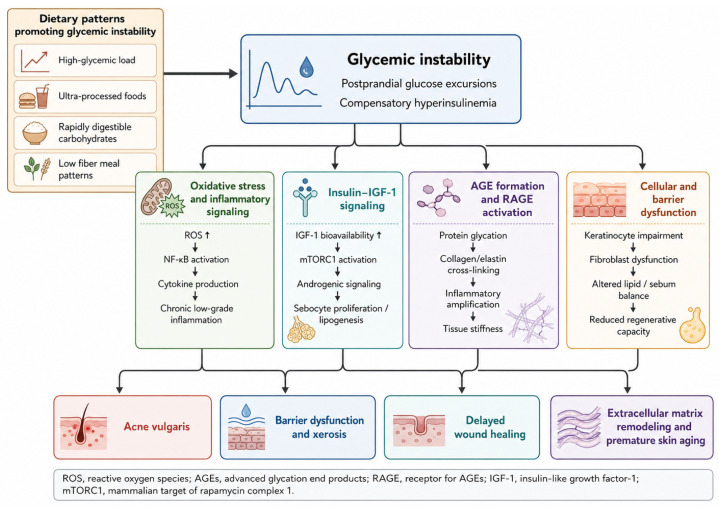
Proposed mechanistic pathways linking glycemic instability with skin dysfunction.

In recent years, increasing attention has been directed toward the biological effects of these dynamic metabolic changes, particularly their relationship with oxidative stress and inflammatory activation [7,8]. The mechanistic pathways presented in Figure 1 summarize the principal biological processes that have been proposed to link glycemic instability with skin dysfunction. These interconnected pathways provide the organizational framework for the following sections, each of which discusses one of the principal mechanisms potentially linking glycemic instability with skin dysfunction. Although presented separately for clarity, these pathways interact extensively and should be regarded as interconnected rather than independent mechanisms. Experimental studies and clinical observations outside dermatology suggest that oscillating glucose exposure may elicit more pronounced oxidative and inflammatory responses than stable hyperglycemia alone. However, whether similar effects occur in cutaneous tissues remains uncertain. These effects have been demonstrated primarily in endothelial cell models and clinical studies investigating vascular complications of diabetes, supporting the biological plausibility that repeated postprandial glucose excursions may contribute to tissue dysfunction through ROS generation and inflammatory pathway activation. Whether these mechanisms operate to a similar extent in cutaneous tissues remains uncertain because direct dermatological evidence is currently limited [20].

Both experimental and clinical studies suggest that repeated glucose fluctuations may contribute to excessive reactive oxygen species (ROS) production and transient activation of pro-inflammatory pathways [21]. Reactive oxygen species have been proposed to represent one of the earliest downstream consequencesof glucose oscillations and may subsequently activate multiple signaling pathways involved in inflammation, extracellular matrix remodeling, and epidermal barrier dysfunction, as illustrated in Figure 1. Oxidative stress associated with impaired glucose regulation has been proposed to involve mitochondrial dysfunction, activation of nuclear factor kappa B (NF-κB), increased cytokine production, and disruption of cellular homeostasis [1,8]. These processes have been proposed to contribute to chronic low-grade inflammation and impaired tissue function. Persistent activation of these pathways has been proposed to create a microenvironment characterized by oxidative damage, altered cellular signaling, and impaired regenerative capacity, thereby providing a mechanistic framework linking metabolic dysregulation with skin dysfunction. Skin cells may be particularly susceptible to metabolic stress, although direct evidence remains limited. Keratinocytes and dermal fibroblasts are highly dependent on metabolic homeostasis for maintaining epidermal barrier integrity, extracellular matrix organization, and regenerative capacity. Therefore, disturbances in glucose regulation may influence skin physiology by affecting cellular signaling, inflammatory responses, and tissue remodeling processes [11,26,27].

Hyperglycemia-associated oxidative stress has been suggested to contribute to impaired keratinocyte proliferation, altered fibroblast activity, extracellular matrix dysfunction, and delayed tissue repair [22,23]. Increased expression of inflammatory mediators, including interleukin-8 and other cytokines involved in cutaneous inflammation, has also been observed under hyperglycemic conditions in keratinocyte models [24]. Taken together, these findings support the hypothesis that oxidative stress represents one of the central mechanisms through which metabolic dysregulation may influence skin physiology. However, because most available evidence originates from experimental models or studies of diabetes mellitus, direct extrapolation to glycemic variability should be interpreted with caution [28].

Although the biological relevance of glycemic variability is increasingly recognized, direct evidence linking CGM-derived measures of glycemic variability with dermatological outcomes remains limited. Most available studies have focused on vascular and metabolic complications of diabetes, whereas relatively few investigations have specifically examined skin physiology or skin disease in relation to glycemic variability. Consequently, the mechanisms discussed in this section should be interpreted as biologically plausible pathways supported primarily by mechanistic and indirect clinical evidence rather than definitive causal relationships. Although glycemic variability represents the central focus of this review, very few studies have directly evaluated CGM-derived metrics such as Time in Range (TIR), Mean Amplitude of Glycemic Excursions (MAGE), Coefficient of Variation (CV), or Continuous Overall Net Glycemic Action (CONGA) in relation to dermatological outcomes. Consequently, current understanding relies predominantly on indirect evidence derived from studies of diabetes mellitus, chronic hyperglycemia, insulin resistance, and dietary glycemic load rather than investigations specifically assessing glycemic variability. Future studies integrating standardized CGM-derived measures with validated dermatological endpoints will be essential to determine whether glycemic variability independently contributes to skin physiology and disease [29,30,31,32].

Glycemic instability, characterized by recurrent postprandial glucose excursions and fluctuations in glucose concentrations, may promote oxidative stress through increased reactive oxygen species (ROS) generation. Oxidative stress has been proposed to activate inflammatory pathways, including NF-κB signaling, contributing to cytokine production, mitochondrial dysfunction, and impaired cellular homeostasis. These processes may further promote the formation of advanced glycation end products (AGEs), extracellular matrix remodeling, epidermal barrier dysfunction, and alterations in insulin/IGF-1 signaling. Together, these interconnected mechanisms have been proposed to contribute to impaired keratinocyte and fibroblast function, altered sebaceous gland activity, reduced skin elasticity, delayed wound healing, and other manifestations of skin dysfunction. Solid arrows indicate proposed mechanistic relationships based primarily on experimental and observational evidence rather than established causal clinical pathways. The pathways are highly interconnected and should not be interpreted as independent biological processes [1,7,8,28,29].

### 3.2. Insulin and IGF-1 Signaling in Skin Dysfunction

Most studies evaluating insulin–IGF-1 signaling have investigated chronic hyperglycemia, insulin resistance, or high-glycemic dietary patterns rather than glycemic variability itself. Therefore, the following mechanisms should be interpreted as indirect evidence supporting a potential role of glycemic fluctuations in skin physiology. Insulin and insulin-like growth factor-1 (IGF-1) play central roles in metabolic regulation and cellular growth and are increasingly recognized as important modulators of skin physiology. Both pathways influence keratinocyte proliferation, sebocyte activity, androgen signaling, and extracellular matrix homeostasis [2,6].

Under conditions of recurrent postprandial hyperglycemia and impaired glucose regulation, compensatory hyperinsulinemia may increase IGF-1 bioavailability and enhance activation of insulin and IGF-1 receptors in cutaneous tissues. Hyperinsulinemia may also reduce hepatic production of insulin-like growth factor-binding proteins (IGFBPs) and sex hormone-binding globulin (SHBG), increasing free androgen availability and amplifying endocrine signaling [6,17].

The insulin–IGF-1 axis has been suggested to be associated with increased sebocyte proliferation, enhanced sebum production, follicular hyperkeratinization, and altered epidermal differentiation. These pathways have been investigated most extensively in acne vulgaris, where elevated IGF-1 signaling has been associated with increased lesion severity and sebaceous gland activity [18]. Experimental studies have also linked insulin and IGF-1 signaling with activation of mammalian target of rapamycin complex 1 (mTORC1), a nutrient-sensitive pathway involved in cellular growth, sebaceous lipogenesis, keratinocyte proliferation, and inflammatory responses implicated in acne pathogenesis [33,34]. In acne vulgaris, dietary interventions reducing glycemic load have been associated with clinical improvement and, in some studies, with reductions in IGF-1 signaling or changes in skin surface lipid composition. These findings support the hypothesis that high-glycemic dietary patterns may modulate sebaceous gland activity through insulin–IGF-1 and nutrient-sensitive pathways, although the magnitude of the effect appears population-dependent [35,36,37,38].

Beyond sebaceous gland activity, chronic hyperinsulinemia may also influence fibroblast function, collagen metabolism, and inflammatory signaling within the skin. Increased insulin and IGF-1 activity has been proposed to interact with oxidative stress pathways and may contribute to tissue remodeling [9].

Despite substantial interest in the insulin–IGF-1 axis, much of the available evidence remains mechanistic or observational in nature. The extent to which insulin-IGF-1 dysregulation independently contributes to individual dermatological conditions remains unclear. Although the insulin–IGF-1 pathway represents a biologically plausible mechanism linking glycemic dysregulation and skin physiology, direct evidence specifically implicating glycemic variability remains limited.

### 3.3. Advanced Glycation End Products (AGEs) and Extracellular Matrix Dysfunction

Advanced glycation end products (AGEs) are generated through non-enzymatic reactions between reducing sugars and proteins, lipids, or nucleic acids. Their formation is accelerated under conditions of chronic hyperglycemia, oxidative stress, and prolonged metabolic dysfunction, resulting in progressive accumulation within tissues characterized by low protein turnover, including the skin [9,10]. Although this review focuses on glycemic variability, most available evidence regarding AGE formation originates from studies of chronic hyperglycemia and diabetes mellitus rather than investigations directly assessing glucose fluctuations. Nevertheless, repeated postprandial glucose excursions have been hypothesized to promote glycation through recurrent transient increases in glucose exposure, although this hypothesis has not yet been confirmed in dermatological studies [9,10].

Within the skin, AGEs accumulate predominantly in long-lived extracellular matrix proteins, particularly collagen and elastin. The accumulation of Maillard reaction products in dermal collagen has been documented in both diabetes mellitus and physiological aging, providing a mechanistic explanation for progressive loss of skin elasticity under conditions of chronic metabolic stress [10,12,13]. Glycation-induced cross-linking has been shown to alter the biomechanical properties of collagen and elastin, resulting in increased tissue stiffness, reduced elasticity, impaired hydration, and diminished regenerative capacity [9,10]. These structural modifications are considered one of the principal mechanisms potentially contributing to accelerated skin aging associated with chronic metabolic dysfunction [9].

Beyond their structural effects, AGEs also exert important biological actions through interaction with the receptor for advanced glycation end products (RAGE). Experimental studies have demonstrated that activation of the AGE–RAGE pathway is associated with increased oxidative stress, activation of nuclear factor kappa B (NF-κB), enhanced production of pro-inflammatory cytokines, and persistent low-grade inflammation [9]. These processes have been proposed to impair fibroblast proliferation, collagen synthesis, extracellular matrix remodeling, and wound healing, thereby compromising skin integrity and regenerative capacity.

Observational studies have associated increased AGE accumulation with several manifestations of cutaneous dysfunction, including reduced skin elasticity, wrinkle formation, xerosis, delayed wound healing, and increased susceptibility to mechanical injury [10,39]. In addition, AGE-induced oxidative and inflammatory signaling may interact with insulin resistance and other metabolic abnormalities, suggesting that glycation represents one component of a broader network linking metabolic dysfunction with skin homeostasis [9,10].

Although the role of AGEs in diabetes-related tissue damage and physiological skin aging is well established, their contribution to dermatological disorders in individuals without overt metabolic disease remains less clear. Likewise, the extent to which AGE accumulation reflects glycemic variability rather than cumulative exposure to chronic hyperglycemia has not yet been determined. Consequently, current evidence supporting a direct role of glycemic variability in AGE-mediated skin dysfunction should be regarded as indirect. Mechanistic studies provide strong biological support for this pathway, whereas clinical evidence specifically evaluating CGM-derived measures of glycemic variability remains limited. Future prospective studies integrating standardized glycemic variability metrics with biomarkers of glycation and validated dermatological outcomes are required to clarify whether glycemic variability independently contributes to AGE-related skin dysfunction.

### 3.4. Skin Barrier Dysfunction and Sebaceous Gland Activity

The epidermal barrier plays an essential role in maintaining skin homeostasis by regulating transepidermal water loss (TEWL), protecting against environmental stressors, and supporting innate immune defense. Proper barrier function depends on coordinated keratinocyte differentiation, epidermal lipid synthesis, sebaceous gland activity, and maintenance of the hydrolipid film covering the skin surface [34].

Although this review focuses on glycemic variability, current knowledge regarding metabolic impairment of epidermal barrier function is derived predominantly from studies of diabetes mellitus and chronic hyperglycemia, whereas direct evidence evaluating the independent contribution of glycemic variability remains limited [19,22,34]. Experimental studies have demonstrated impaired epidermal barrier recovery under diabetic conditions, while investigations of diabetic xerosis suggest that altered keratinocyte differentiation, reduced skin hydration, and disrupted lipid organization contribute to increased transepidermal water loss and skin fragility [19,22,30,34].

Mechanistic studies have proposed several pathways through which disturbances in glucose metabolism may impair epidermal barrier integrity, including oxidative stress, chronic low-grade inflammation, altered lipid metabolism, and impaired cellular regeneration. Under hyperglycemic conditions, impaired keratinocyte differentiation and disrupted epidermal homeostasis have been associated with increased transepidermal water loss, xerosis, greater susceptibility to irritation, and microdamage [22,30,34]. However, these findings originate primarily from experimental models and diabetic populations rather than studies directly evaluating glycemic variability.

Sebaceous gland activity also appears to be influenced by metabolic and hormonal signaling. Hyperinsulinemia and increased IGF-1 activity may stimulate sebocyte proliferation and enhance sebum production through androgen-dependent and nutrient-sensitive pathways [6,25,33]. In addition, glycemic dysregulation has been hypothesized to influence sebum composition by increasing oxidation-prone lipid fractions while reducing components involved in barrier stability and antimicrobial protection, although direct evidence supporting these effects remains limited.

Collectively, these alterations may potentially contribute to seborrheic skin features, follicular obstruction, inflammatory acne lesions, impaired epidermal resilience, skin dryness, and altered barrier function. Barrier dysfunction associated with metabolic dysregulation has also been hypothesized to facilitate microbial colonization and amplify cutaneous inflammatory responses, thereby potentially contributing to the development or persistence of inflammatory skin disorders.

Overall, available evidence provides biologically plausible mechanisms linking metabolic dysregulation with impaired epidermal barrier function. However, most data derive from mechanistic studies, experimental models, or investigations of diabetes mellitus and chronic hyperglycemia. Direct clinical evidence demonstrating that CGM-derived measures of glycemic variability independently influence epidermal barrier integrity or sebaceous gland function remains scarce. Future prospective studies integrating standardized glycemic variability metrics with objective assessments of barrier function, such as TEWL and skin hydration, are needed to clarify these relationships.

### 3.5. Metabolic Stress and Extracellular Matrix Remodeling

Maintenance of skin structure and mechanical integrity depends on continuous remodeling of the extracellular matrix (ECM), a process regulated primarily by dermal fibroblasts and involving collagen, elastin, hyaluronic acid, proteoglycans, and other structural components responsible for skin elasticity, hydration, and regenerative capacity. Although this review focuses on glycemic variability, evidence linking extracellular matrix remodeling specifically to glycemic variability remains scarce. Most mechanistic insights derive from studies of chronic hyperglycemia, diabetes mellitus, or aging-associated glycation and should therefore be regarded as indirect evidence [30,31].

Experimental and observational studies have proposed that chronic metabolic dysregulation may impair extracellular matrix remodeling through interconnected oxidative, inflammatory, and glycation-related mechanisms. Persistent hyperglycemia has been associated with altered fibroblast activity, reduced collagen synthesis, impaired cellular migration, premature cellular senescence, and disturbances in extracellular matrix homeostasis [9,10].

Oxidative stress has been proposed to play an important role in these processes. Increased reactive oxygen species (ROS) production may impair mitochondrial function and activate pathways involved in extracellular matrix degradation, including matrix metalloproteinase (MMP) activation and collagen fragmentation. In parallel, chronic inflammatory signaling may suppress fibroblast regenerative capacity, impair collagen synthesis, and delay tissue repair, thereby contributing to progressive deterioration of dermal structure.

Advanced glycation end products (AGEs) have also been proposed to contribute to extracellular matrix dysfunction through irreversible cross-linking of collagen and elastin fibers. These structural modifications reduce tissue flexibility, impair normal matrix turnover, and have been associated with increased skin stiffness, reduced elasticity, wrinkle formation, and diminished regenerative capacity [9,10]. Similar mechanisms have been described in diabetic wound healing, where impaired fibroblast function and abnormal extracellular matrix remodeling have been associated with delayed tissue repair and chronic wound persistence [40].

In addition to collagen and elastin remodeling, metabolic dysregulation may also influence the production of extracellular matrix-associated molecules involved in tissue hydration and structural organization, including hyaluronic acid and proteoglycans. Collectively, these mechanisms may contribute to age-related alterations in dermal architecture and impaired skin function.

Although extracellular matrix dysfunction is well documented in diabetes-related tissue damage and physiological skin aging, the specific contribution of glycemic variability remains uncertain. Most available evidence derives from mechanistic studies and investigations of chronic hyperglycemia rather than studies directly evaluating glycemic variability. Consequently, current evidence supports biologically plausible mechanisms linking metabolic dysregulation with extracellular matrix remodeling, but direct clinical evidence demonstrating an independent effect of glycemic variability remains limited. Future prospective studies incorporating standardized CGM-derived measures together with objective assessments of extracellular matrix integrity are needed to clarify these relationships.

## 4. Dietary Patterns and Determinants of Glycemic Instability

### 4.1. High-Glycemic Diets and Postprandial Glucose Excursions

Dietary patterns characterized by frequent consumption of high-glycemic-index (GI) and highly processed foods may promote postprandial glucose excursions and broader metabolic instability. High-GI foods are rapidly digested and absorbed, leading to abrupt elevations in blood glucose concentrations followed by compensatory insulin responses. Recurrent postprandial glucose elevations have been associated with hyperinsulinemia, oxidative stress, and activation of inflammatory pathways implicated in metabolic dysfunction [16,41].

In contrast, low-glycemic dietary patterns are associated with slower glucose absorption and may contribute to reduced postprandial glycemic variability and lower insulin demand. Foods rich in dietary fiber, minimally processed whole grains, legumes, and vegetables may contribute to greater metabolic stability by delaying gastric emptying and attenuating rapid glucose excursions [42].

The potential dermatological relevance of high-glycemic diets has been investigated most extensively in acne vulgaris. Several studies have suggested that dietary patterns with elevated glycemic load are associated with increased insulin and IGF-1 signaling, sebaceous gland activity, and acne severity [17,33]. At the same time, available evidence remains heterogeneous and is influenced by multiple confounding factors, including obesity, hormonal status, lifestyle, and overall dietary quality.

Importantly, glycemic response appears to be shaped by broader dietary context rather than individual nutrients alone. Together, these findings suggest that glycemic response is shaped by broader dietary context rather than individual nutrients alone.

### 4.2. Meal Composition, Food Order, and Glycemic Modulation

Postprandial glycemic response depends not only on carbohydrate intake itself, but also on meal composition, nutrient interactions, and the order in which foods are consumed. These factors may substantially modify postprandial glucose and insulin responses and thereby influence short-term metabolic variability [43].

Consumption of dietary fiber, protein, and fats before carbohydrate-rich foods has been associated with attenuation of postprandial glucose elevations and lower insulin demand. This effect is likely related to delayed gastric emptying, slower glucose absorption, and modulation of incretin signaling. As a result, meal sequencing strategies may reduce the amplitude of postprandial glycemic fluctuations and potentially influence postprandial metabolic responses [42,43,44].

Dietary fiber appears to play a role in glycemic modulation. Soluble fiber fractions may slow intestinal glucose absorption and reduce postprandial glycemic variability. In addition, fermentation of resistant starch and fiber-derived substrates by gut microbiota leads to production of short-chain fatty acids with potential anti-inflammatory and metabolically beneficial effects [42]. In contrast, meals dominated by rapidly digestible carbohydrates and low fiber content may promote abrupt glucose excursions and compensatory hyperinsulinemia.

Meal composition has been associated with changes in endocrine and inflammatory responses relevant to skin physiology. Diets characterized by high intake of ultra-processed foods and refined carbohydrates have been associated with oxidative stress, chronic low-grade inflammation, and alterations in insulin–IGF-1 signaling pathways that may be involved in sebaceous gland activity and skin aging processes [16,41].

Direct evidence linking meal sequencing and postprandial glycemic modulation with dermatological outcomes remains limited, and most available studies have focused primarily on metabolic rather than skin-specific endpoints.

### 4.3. Physical Activity and Glycemic Stability

Physical activity is one of the major non-pharmacological factors influencing glucose metabolism and postprandial glycemic regulation. Regular exercise improves insulin sensitivity and is associated with enhanced skeletal muscle glucose uptake and may reduce postprandial glucose excursions, thereby supporting greater metabolic stability [44].

Both aerobic and resistance exercise have been associated with improved glycemic control through insulin-dependent and insulin-independent mechanisms. Muscle contraction stimulates translocation of glucose transporter type 4 (GLUT4) to the cell membrane, facilitating glucose uptake independently of insulin signaling and contributing to improved glucose homeostasis during metabolic stress [5,44].

Regular exercise may also influence inflammatory and oxidative pathways relevant to skin physiology. Improved insulin sensitivity and reduced chronic low-grade inflammation may indirectly support epidermal barrier function, tissue regeneration, extracellular matrix maintenance, and microcirculation, all of which are important for maintaining skin integrity and wound healing.

Despite well-established metabolic benefits of physical activity, direct evidence linking exercise-induced glycemic stabilization with dermatological outcomes remains limited. Most available studies have focused primarily on systemic metabolic parameters rather than skin-specific effects.

## 5. Clinical Implications for Dermatological Disorders

Disturbances in glucose metabolism may be associated with multiple aspects of skin physiology through mechanisms involving oxidative stress, chronic inflammation, hormonal dysregulation, extracellular matrix dysfunction, and impaired epidermal barrier function. Many of these pathways have been studied extensively in diabetes mellitus and insulin resistance, although similar mechanisms may also contribute to skin dysfunction in broader metabolic contexts.

The skin is particularly sensitive to chronic metabolic stress because of its high cellular turnover, constant environmental exposure, and dependence on tightly regulated regenerative and immunological processes. As a result, disturbances associated with impaired glucose regulation may be associated with inflammatory skin disease, tissue repair, barrier integrity, sebaceous gland activity, and aging-related structural changes.

Current evidence linking glycemic dysregulation with dermatological disorders varies considerably depending on the specific condition investigated. The following sections summarize current mechanistic and clinical findings related to selected inflammatory, degenerative, and regenerative skin disorders.

### 5.1. Acne Vulgaris

Acne vulgaris is one of the most common chronic inflammatory skin disorders and involves sebaceous gland hyperactivity, follicular hyperkeratinization, microbial dysbiosis, and altered inflammatory responses. Although acne pathogenesis is multifactorial and influenced by genetic, hormonal, environmental, and lifestyle-related factors, disturbances in glucose metabolism may also be associated with disease severity through mechanisms involving hyperinsulinemia, IGF-1 signaling, and nutrient-sensitive inflammatory pathways. Evidence linking glycemic dysregulation with acne is supported by mechanistic studies, observational investigations, and several randomized dietary intervention trials, although direct studies evaluating glycemic variability remain limited [17,33].

Dietary patterns characterized by high glycemic load may promote rapid postprandial glucose excursions and compensatory insulin secretion. Elevated insulin concentrations may increase IGF-1 bioavailability while reducing levels of insulin-like growth factor-binding proteins (IGFBPs) and sex hormone-binding globulin (SHBG), leading to enhanced androgen signaling and increased sebaceous gland activity [6]. Increased IGF-1 activity has also been linked to activation of mammalian target of rapamycin complex 1 (mTORC1), a nutrient-sensitive pathway involved in sebocyte proliferation, lipogenesis, keratinocyte growth, and inflammatory responses implicated in acne pathogenesis [33].

Several observational studies and dietary intervention trials have reported associations between low-glycemic dietary patterns and improvement in acne severity. In a randomized controlled trial, individuals following a low-glycemic-load diet demonstrated reductions in inflammatory acne lesions, sebaceous gland size, and markers associated with sebocyte activity compared with participants consuming a conventional high-glycemic diet. Similar findings have been reported in studies investigating dietary interventions aimed at improving insulin sensitivity and postprandial glucose regulation [17].

Oxidative stress and chronic low-grade inflammation associated with impaired glucose regulation have been suggested to further contribute by amplifying inflammatory signaling within pilosebaceous units. Hyperglycemia-associated oxidative stress has been linked to increased cytokine production, altered lipid metabolism, and enhanced inflammatory activity in skin cells, potentially contributing to lesion persistence and inflammatory activity [32].

Despite growing interest in the relationship between glycemic dysregulation and acne, available evidence remains influenced by multiple confounding factors, including obesity, hormonal status, stress, sleep quality, and overall dietary patterns. In addition, many studies remain observational or involve relatively small study populations, making causal interpretation difficult.

Overall, the acne-related evidence appears stronger than for most other dermatological outcomes discussed in this review. Randomized dietary trials, observational studies, and systematic reviews suggest that high-glycemic-index/load diets may have a modest pro-acnegenic effect, plausibly mediated by insulin–IGF-1 signaling, mTORC1 activation, sebaceous lipogenesis, and follicular hyperkeratinization. However, available studies remain heterogeneous, and dietary effects should be interpreted as modulatory rather than independently causal [17,36,37,38,45].

### 5.2. Hidradenitis Suppurativa

Hidradenitis suppurativa (HS) is a chronic, recurrent inflammatory skin disease characterized by painful nodules, abscesses, sinus tract formation, and progressive scarring affecting intertriginous regions [39,46]. In recent years, growing evidence has demonstrated that HS is frequently accompanied by metabolic comorbidities, including obesity, metabolic syndrome, insulin resistance, and type 2 diabetes mellitus. Recent systematic reviews and meta-analyses have further confirmed that individuals with HS exhibit a significantly higher prevalence of diabetes compared with the general population, emphasizing the close relationship between chronic cutaneous inflammation and systemic metabolic dysfunction [46,47].

The biological mechanisms underlying this association appear to be multifactorial. Chronic low-grade inflammation, oxidative stress, adipose tissue dysfunction, and insulin resistance have all been implicated in the pathogenesis of HS and may collectively contribute to sustained activation of inflammatory pathways. Dysregulated glucose metabolism has been proposed to amplify inflammatory responses through increased production of pro-inflammatory cytokines, activation of NF-κB signaling, and enhanced oxidative stress, mechanisms that have also been discussed throughout this review as potential consequences of glycemic dysregulation. These overlapping pathways suggest that metabolic abnormalities may influence HS severity indirectly by promoting a systemic pro-inflammatory environment rather than acting as independent causal factors [48].

Although epidemiological evidence consistently supports an association between HS and diabetes mellitus, considerably less is known about the potential contribution of glycemic variability itself [46]. Most available studies have evaluated chronic hyperglycemia or diabetes status, whereas direct assessment of dynamic glucose fluctuations using continuous glucose monitoring (CGM) has not yet been performed in patients with HS. Consequently, it remains uncertain whether repeated postprandial glucose excursions independently influence disease activity, inflammatory burden, or clinical progression beyond the effects attributable to chronic metabolic dysfunction. Given that oxidative stress, inflammatory signaling, insulin resistance, and advanced glycation end product (AGE) formation have all been implicated in both glycemic dysregulation and HS pathogenesis, this disease represents a particularly relevant model for future investigation. Studies incorporating standardized CGM-derived metrics, including Time in Range (TIR), coefficient of variation (CV), and mean amplitude of glycemic excursions (MAGE), may help clarify whether glycemic instability contributes to HS pathophysiology and could identify novel metabolic targets for disease prevention and management [20,21,48]. At present, however, such hypotheses remain biologically plausible but are supported predominantly by mechanistic and epidemiological evidence rather than direct clinical investigations.

### 5.3. Delayed Wound Healing in Diabetes and Metabolic Dysregulation

Wound healing requires coordinated interactions between inflammatory cells, keratinocytes, fibroblasts, endothelial cells, and extracellular matrix components. These processes are highly energy-dependent and closely regulated by metabolic homeostasis. Disturbances in glucose metabolism, particularly chronic hyperglycemia, have been associated with impairment of multiple stages of tissue repair and chronic wound formation. Evidence supporting metabolic influences on wound healing derives predominantly from diabetes research and experimental models rather than studies specifically evaluating glycemic variability [10,40].

Hyperglycemia-associated oxidative stress and chronic inflammation are widely regarded as important contributors to impaired regenerative capacity in diabetic and metabolically dysregulated tissues. Excessive reactive oxygen species (ROS) production may disrupt mitochondrial function, impair cellular migration, and reduce fibroblast and keratinocyte activity, which has been linked to impaired tissue regeneration. Chronic inflammation may also prolong the inflammatory phase and impair transition to proliferative and remodeling stages of wound healing [40].

Experimental evidence suggests that high-glucose environments may be associated with altered keratinocyte behavior and cytokine secretion. Lan et al. [32] demonstrated that hyperglycemic conditions increased oxidative stress and interleukin-8 (IL-8) secretion in keratinocytes, supporting the concept that glucose dysregulation may directly influence inflammatory and regenerative processes within the skin.

Glycation-related extracellular matrix dysfunction may further impair tissue repair. AGE accumulation has been shown to contribute to abnormal collagen cross-linking, reduced matrix flexibility, impaired angiogenesis, and altered fibroblast activity, contributing to impaired wound closure and abnormal tissue remodeling [9,10].

Microvascular dysfunction associated with insulin resistance and chronic metabolic stress may additionally reduce tissue perfusion and oxygen delivery, further compromising regenerative processes. These mechanisms are particularly relevant in diabetic wound healing, where impaired circulation, neuropathy, chronic inflammation, and metabolic dysregulation collectively contribute to chronic ulcers and persistent tissue injury.

Although delayed wound healing is strongly associated with diabetes mellitus and chronic hyperglycemia, the specific role of glycemic variability remains less clearly established. Most available evidence is derived from diabetic populations, while direct studies investigating postprandial metabolic instability and skin regeneration remain limited.

### 5.4. Glycemic Dysregulation and Skin Aging

Skin aging is a multifactorial process influenced by intrinsic aging, environmental exposure, hormonal changes, oxidative stress, and metabolic factors. Metabolic dysregulation may be associated with accelerated structural and functional skin aging through mechanisms involving oxidative stress, chronic inflammation, extracellular matrix dysfunction, and accumulation of advanced glycation end products (AGEs). Current evidence consists primarily of mechanistic studies and observational investigations, whereas direct clinical evidence relating glycemic variability to skin aging is lacking [9,10].

One of the most well-described mechanisms linking metabolic dysfunction with skin aging is non-enzymatic glycation of long-lived structural proteins such as collagen and elastin. AGE accumulation has been shown to promote irreversible cross-linking of extracellular matrix fibers, leading to increased tissue stiffness, reduced elasticity, impaired hydration, and diminished regenerative capacity. These alterations are thought to contribute to wrinkle formation, loss of skin firmness, and progressive deterioration of dermal architecture [9].

Oxidative stress associated with chronic hyperglycemia and impaired glucose regulation may further accelerate cellular aging processes by disrupting mitochondrial function and promoting inflammatory signaling. Increased reactive oxygen species (ROS) production has been linked to fibroblast dysfunction, reduced collagen synthesis, activation of matrix metalloproteinases (MMPs), and extracellular matrix degradation associated with cutaneous aging [10].

Several clinical observations have also suggested associations between glycemic markers and perceived skin aging. The study reported relationships between biochemical markers of glucose metabolism and perceived facial age, supporting the concept that metabolic status may influence visible aging characteristics. Similar associations have been described in studies investigating insulin resistance, metabolic syndrome, and structural skin changes [43].

Metabolic dysregulation may also impair epidermal barrier integrity and tissue hydration, contributing to skin dryness, reduced resilience, and greater susceptibility to environmental stressors. Chronic low-grade inflammation associated with metabolic dysfunction may additionally amplify aging-related changes through persistent activation of inflammatory and oxidative pathways. Experimental studies provide strong mechanistic support for the involvement of oxidative stress and glycation in skin aging. Observational studies have also reported associations between markers of glucose metabolism and perceived skin aging. However, direct clinical investigations specifically evaluating glycemic variability or CGM-derived measures in relation to skin aging remain lacking. Therefore, current evidence supports biological plausibility rather than a confirmed causal relationship.

Although mechanisms linking hyperglycemia and glycation with tissue aging are well established, the specific contribution of glycemic variability to premature skin aging remains unclear. Most available evidence is derived from mechanistic studies and indirect clinical observations rather than direct clinical investigations.

### 5.5. Xerosis and Barrier Dysfunction

Xerosis is one of the most frequently reported cutaneous manifestations associated with diabetes mellitus and chronic metabolic dysfunction. Clinically, it is characterized by skin dryness, roughness, scaling, reduced elasticity, and increased susceptibility to irritation and microdamage. Most available evidence derives from diabetic populations and experimental hyperglycemia models [34].

Several mechanisms may contribute to impaired skin hydration under conditions of chronic hyperglycemia and metabolic dysregulation. Oxidative stress and impaired keratinocyte differentiation may disrupt maintenance of the epidermal barrier, leading to increased transepidermal water loss (TEWL) and reduced epidermal hydration [22]. Glycation-related alterations in extracellular matrix proteins may additionally reduce skin elasticity and impair water retention capacity.

Microvascular dysfunction associated with insulin resistance and chronic metabolic stress may further impair nutrient and oxygen delivery to the skin and compromise regenerative processes. Altered sebaceous gland activity and changes in skin surface lipid composition may be associated with destabilization of epidermal homeostasis and increased susceptibility to environmental stressors and mechanical injury.

Barrier dysfunction associated with xerosis may facilitate microbial colonization and contribute to low-grade cutaneous inflammation. Increased skin dryness has also been associated with pruritus, impaired wound healing, and elevated susceptibility to skin infections in metabolically dysregulated individuals [34].

Although xerosis commonly coexists with diabetes and chronic metabolic disorders, evidence directly linking glycemic variability with epidermal barrier impairment remains limited. Most available data concern chronic hyperglycemia and diabetes-related skin complications rather than dynamic glucose fluctuations.

### 5.6. Atopic Dermatitis

Atopic dermatitis (AD) is a chronic inflammatory skin disorder characterized by impaired epidermal barrier function, immune dysregulation, pruritus, and recurrent eczematous lesions. Although AD pathogenesis is driven primarily by genetic, immunological, and barrier-related mechanisms, recent studies have explored whether metabolic disturbances may influence selected aspects of epidermal function and inflammatory activity in AD [49].

Impaired epidermal barrier function in AD has been associated with oxidative stress and inflammatory signaling, mechanisms that partially overlap with pathways involved in metabolic dysregulation. Alterations in glucose transport and local glucose metabolism within the skin have also been proposed as factors potentially influencing epidermal homeostasis and inflammatory responses in AD [49].

Increased sweat glucose concentrations and altered expression of glucose transporter type 2 (GLUT2) in individuals with atopic dermatitis, suggesting disturbances in local glucose handling within the skin. These observations suggest a possible association between local glucose metabolism and epidermal inflammation in AD, although the clinical significance of these findings remains uncertain. Direct evidence linking glycemic variability with AD pathogenesis remains scarce [49].

Dietary interventions aimed at reducing inflammatory burden and improving metabolic regulation have also been investigated in selected AD populations. However, available evidence remains inconsistent, and relationships between glycemic regulation, dietary glycemic load, and AD severity have not been clearly established. Many studies are limited by small study populations, heterogeneous interventions, and substantial confounding factors.

Overall, current evidence does not support a direct causal relationship between glycemic instability and atopic dermatitis. Available findings remain largely indirect and require confirmation in larger mechanistic and clinical studies.

### 5.7. Psoriasis

Psoriasis is a chronic immune-mediated inflammatory skin disease that is increasingly recognized as a systemic disorder associated with obesity, metabolic syndrome, insulin resistance, and type 2 diabetes mellitus. These metabolic comorbidities suggest that disturbances in glucose homeostasis may contribute to systemic inflammatory processes relevant to psoriasis pathogenesis. Current evidence is largely observational and focuses on metabolic comorbidity rather than glycemic variability itself [43].

Several shared mechanisms have been proposed to link psoriasis with metabolic dysfunction. Chronic low-grade inflammation, oxidative stress, and insulin resistance appear to promote reciprocal interactions between metabolic and immune pathways, creating a bidirectional relationship in which metabolic abnormalities may exacerbate inflammatory activity while psoriasis itself may increase the risk of developing metabolic disease. In particular, the IL-23/IL-17 inflammatory axis, a key driver of psoriasis, has also been implicated in insulin resistance and impaired glucose metabolism, providing a biologically plausible connection between glucose dysregulation and psoriatic inflammation [50].

Current evidence also suggests that patients with psoriasis have an increased prevalence of type 2 diabetes and other components of the metabolic syndrome compared with the general population. Lifestyle interventions aimed at improving metabolic health, including weight reduction and optimization of glycemic control, may provide additional clinical benefits in selected patients. However, the independent contribution of dietary glycemic index or glycemic load to psoriasis severity remains uncertain, and available evidence is influenced by multiple confounding factors, including obesity, overall dietary quality, and physical activity. Although dietary interventions have been investigated as adjunctive strategies in psoriasis management, the independent effects of dietary glycemic index or glycemic load remain insufficiently established, and current evidence suggests that overall metabolic improvement and weight reduction are likely to play a greater role than glycemic modulation alone [50].

To date, no clinical studies have directly evaluated CGM-derived measures of glycemic variability in relation to psoriasis severity or treatment response. Therefore, the potential contribution of postprandial glucose excursions should currently be regarded as biologically plausible but supported primarily by mechanistic and observational evidence rather than direct clinical investigations.

## 6. Current Limitations and Future Directions

Despite increasing interest in the relationship between metabolic dysregulation and skin health, available evidence remains limited and methodologically heterogeneous. Most published studies focus on chronic hyperglycemia and diabetes-related complications, whereas the specific effects of glycemic variability and postprandial glucose excursions on skin physiology remain less thoroughly investigated. The available evidence differs substantially across study designs. Mechanistic studies provide important biological insights but cannot establish clinical relevance. Observational studies identify associations but remain susceptible to confounding. Randomized clinical trials evaluating glycemic variability and dermatological outcomes are scarce. Consequently, many of the mechanisms discussed throughout this review should be interpreted as biologically plausible hypotheses rather than established causal pathways.

Much of the current literature is based on observational studies, mechanistic experiments, or relatively small clinical interventions, making causal interpretation difficult. In addition, many studies are influenced by confounding factors such as obesity, hormonal status, lifestyle, and overall dietary quality, all of which may independently affect both metabolic health and skin physiology. Importantly, most mechanistic interpretations in this field should not be interpreted as evidence of causality but rather as biologically plausible associations.

Another important limitation is the lack of standardized methods for assessing glycemic variability in dermatological research. Most available studies rely on indirect metabolic markers, including fasting glucose levels, insulin resistance indices, or self-reported dietary habits, rather than continuous glucose monitoring (CGM)-based approaches that more accurately capture dynamic glucose changes. Consequently, the specific contribution of postprandial glycemic instability to skin dysfunction remains insufficiently characterized.

The strength of available evidence appears to differ substantially between dermatological conditions. Associations between glycemic regulation and acne vulgaris are supported by both mechanistic and clinical observations involving insulin–IGF-1 signaling and dietary glycemic load. In contrast, evidence linking glycemic instability with atopic dermatitis, xerosis, epidermal barrier dysfunction, or premature skin aging remains more limited and largely indirect.

Future studies should prioritize longitudinal and interventional designs evaluating dermatological outcomes alongside metabolic parameters. Greater use of continuous glucose monitoring, skin-specific biomarkers, and standardized dermatological endpoints may help clarify the clinical relevance of glycemic instability in skin physiology and disease progression.

Overall, current evidence linking glycemic variability with dermatological outcomes remains largely indirect and is primarily derived from mechanistic and metabolic studies, with limited integration of CGM-based assessments in dermatological research.

## 7. Conclusions

Disturbances in glucose homeostasis may be associated with skin physiology through mechanisms involving oxidative stress, chronic inflammation, insulin–IGF-1 signaling, glycation-related tissue damage, and extracellular matrix dysfunction. These processes have been proposed to influence epidermal barrier integrity, sebaceous gland activity, tissue repair, and skin aging.

Current evidence appears strongest for acne vulgaris and diabetes-associated impairment of wound healing, whereas associations involving atopic dermatitis, xerosis, and premature skin aging remain less clearly established and are based mainly on indirect or mechanistic observations.

Current evidence supports biologically plausible associations between metabolic dysregulation and multiple aspects of skin physiology. However, most available evidence derives from mechanistic experiments, observational studies, or investigations of chronic hyperglycemia rather than studies directly evaluating glycemic variability. Consequently, causal relationships between glycemic variability and dermatological outcomes cannot yet be established. Future prospective studies integrating CGM-derived metrics with standardized dermatological endpoints are required to determine whether glycemic variability independently influences skin health.

## Data Availability

No new data were created or analyzed in this study. Data sharing is not applicable to this article.
